# The Differences of Serum Metabolites Between Patients With Early-Stage Alzheimer's Disease and Mild Cognitive Impairment

**DOI:** 10.3389/fneur.2019.01223

**Published:** 2019-11-22

**Authors:** Wei-Chieh Weng, Wen-Yi Huang, Hsiang-Yu Tang, Mei-Ling Cheng, Kuan-Hsing Chen

**Affiliations:** ^1^Department of Neurology, College of Medicine, Keelung Chang Gung Memorial Hospital, Chang Gung University, Keelung, Taiwan; ^2^Department of Biomedical Sciences, College of Medicine, Chang Gung University, Taoyuan, Taiwan; ^3^Healthy Aging Research Center, Chang Gung University, Taoyuan, Taiwan; ^4^Metabolomics Core Laboratory, Chang Gung University, Taoyuan, Taiwan; ^5^Clinical Metabolomics Core Laboratory, Linkou Chang Gung Memorial Hospital, Taoyuan, Taiwan; ^6^Kidney Research Center, College of Medicine, Linkou Chang Gung Memorial Hospital, Chang Gung University, Taoyuan, Taiwan; ^7^Kidney Research Center, College of Medicine, Linkou Chang Gung Memorial Hospital, Chang Gung University, Taoyuan, Taiwan

**Keywords:** Alzheimer's disease, mild cognitive impairment, dementia, metabolomics, P 180 kit, serum metabolite

## Abstract

**Background:** Mild cognitive impairment (MCI) is regarded as a transition phase between normal aging and Alzheimer's disease (AD). Identification of novel and non-invasive biomarkers that can distinguish AD at an early stage from MCI is warranted for therapeutic and support planning. The goal of this study was to identify the differences of serum metabolomic profiles between MCI and early-stage AD, which could be potential non-invasive biomarkers for early diagnosis of AD.

**Methods:** The subjects enrolled in the study were classified into two diagnostic groups: MCI (*n* = 40) and early-stage AD (*n* = 40). Targeted metabolomics analysis of serum samples was performed using the Biocrates Absolute-IDQ P180 kit. Targeted metabolic data were analyzed by TargetLynx, and MetIDQ software was applied to integrate the metabolites by automated calculation of metabolite concentrations.

**Results:** The datasets of targeted metabolite analysis were analyzed by the orthogonal-projection-to-latent-structure–discriminant-analysis (OPLS-DA) model. The OPLS-DA score plots demonstrated considerable separation between the MCI and early-stage AD patients. The levels of pimelylcarnitine, putrescine, SM (OH) C24:1, and SM C24:0 were significantly lower, whereas the levels of acetylornithine, methionine sulfoxide, and PC ae C44:3 were significantly higher in early-stage AD patients as compared with MCI patients. Receiver operating characteristic curve analysis of a combination of three lipid metabolites [SM (OH) C24:1, SM C24:0, and PC ae C44:3] showed an acceptable discrimination between the early-stage AD and MCI patients (area under the curve = 0.788).

**Conclusions:** Our results characterized the differences of serum metabolic profiles between MCI and early-stage AD patients. The positive findings from this study indicate that the minimally invasive method of blood sampling may help to identify patients with AD at an early stage from those with MCI.

## Introduction

Alzheimer's disease (AD) is a neurodegenerative disease of the brain, characterized by senile plaques, neurofibrillary tangles, selective loss of neurons and synapse, inflammation and glial responses, and vascular alterations (1). The clinical features of AD include memory impairment, cognitive decline, and behavior changes (2). Mild cognitive impairment (MCI) is regarded as a transition period between age-appropriate memory changes and AD (3). The presentation of MCI includes cognitive deficits, mainly in memory functions, with preservations of independence in everyday activities and does not fulfill the criteria of AD, other dementia disorders, or other mental diseases. MCI is associated with an increased risk of progress to AD (4). The outcomes of the MCI state are diverse and with several possibilities, which even include improving back to normal cognition (5).

Recent research has focused on searching for sensitive and specific biomarkers for the early diagnosis of AD and for identifying MCI patients who will progress to AD from stable MCI or healthy elderly control subjects (6–12). The ideal biomarkers of AD should be non-invasive and could reflect the disease-related biological processes, such as blood samples. Other non-invasive biomarker studies for AD have been done in saliva (13, 14) or tear fluid (15). However, the comparisons of saliva and serum analyses revealed the limitations in using saliva for biomarker discovery (16). There is growing evidence that a single biomarker cannot have enough sensitivity and specificity for AD diagnosis since different research groups identified different biomarkers, and some results cannot be reproduced (6, 17, 18). Therefore, the ideal non-invasive biomarkers for detecting AD in very early stage remain uncertain.

Metabolomics is a discipline specially used in the global study of small molecules in cells, tissues, and biofluids. Concentration changes of specific groups of metabolites may reflect the state of disease progression, and metabolomics has become a powerful tool for biomarker development (10). Currently, technologies of metabolomics have allowed comprehensive and quantitative investigation of different metabolites in various diseases (19). A systemic review by Jiang et al. demonstrated that the concentrations of several metabolites, including lipids (higher phosphatidylcholines, sphingomyelins (SMs), and lysophosphatidylcholine and lower docosahexaenoic acid and high-density lipoprotein subfractions), amino acids (lower branched-chain amino acids, taurine, and higher glutamate, glutamine, and anthranilic acid), were associated with cognitive decline and the incidence or progression of dementia (20). In addition, Fleszar et al. showed that l-arginine/NO pathway in blood was altered in AD and vascular dementia (21). And dysregulated arginine metabolism in urine may serve as a diagnostic biomarker for old MCI patients (22). Moreover, Kim et al. reported that primary fatty amides in plasma were associated with brain amyloid burden, hippocampal volume, and memory (23).

The aim of the present study was to analyze the metabolome of serum samples from MCI and early-stage AD patients. We quantitatively analyzed 40 acylcarnitine metabolites, 21 amino acids, 19 biogenic amines, 15 sphingolipids, and 90 glycerophospholipids using the Absolute-IDQ P180 kit (Biocrates Life Sciences AG, Innsbruck, Austria). We here demonstrated that the concentrations of several serum metabolites were significantly different between patients of MCI and early-stage AD, which may serve as a potential non-invasive biomarker for distinguishing MCI patients who will progress to early-stage AD from MCI patients who will remain in the MCI stage.

## Materials and Methods

### Participants

Patients who visited an outpatient clinic of neurology for memory impairment or behavior change from July 2015 to September 2017 were potential participants of this study. Evaluation of medical history and physical and neurological examinations were performed by two experienced neurologists. Blood tests for a dementia survey that included cortisol levels, thyroid function, syphilis screen, vitamin B12, and folate level and a brain imaging study that included computed tomography (CT) or magnetic resonance imaging (MRI) were arranged as routine survey for dementia. Patients with abnormalities in blood tests for the dementia survey were excluded. The brain imaging study was evaluated by an experienced neuroradiologist, and those with abnormal brain lesions such as old cerebrovascular insults, tumor, encephalomalacia, marked white matter lesions, or other brain lesions were excluded from this study. Patients with a history of psychiatric disorder, depression, old stroke, severe renal or liver dysfunction, or malignancy were also excluded. Mini-Mental State Examination (MMSE) (24), Clinical Dementia Rating (CDR) (25), and Cognitive Abilities Screening Instrument (CASI) (26) were used for dementia evaluation and were performed by an experienced neuropsychologist. Patients were excluded from the study if (1) age is <65 years; (2) they received education for <6 years; (3) they had abnormality in the blood test for the dementia survey that included cortisol levels, thyroid functions, syphilis screen, vitamin B12, and folate levels; (4) CT or MRI show abnormal brain lesions such as old cerebrovascular insults, tumor, encephalomalacia, marked white matter lesions, or other brain lesions; (5) they had a history of psychiatric disorder, depression, old stroke, or malignancy; (6) they had abnormality of renal or liver function tests; (7) they had poorly controlled diabetes mellitus (glycohemoglobin ≧ 7%); (8) they had an MMSE score of 30 or <20 and a CDR score of 0 or >1; (9) they already used a cholinesterase inhibitor. To reduce the influence of hypnotic drugs on metabolomics profiles in these patients, we collected blood samples from the patients after prohibiting eatables and medication intake for at least 8 h in our study.

MCI was diagnosed using the criteria originally proposed by the Mayo Clinic Alzheimer's Disease Research Center (27). The MCI criteria were as follows: (1) memory complaint by patient, family, or physician; (2) normal activities of daily living; (3) normal global cognitive function; (4) objective impairment in memory or in one other area of cognitive function; (5) a CDR score of 0.5 and an MMSE score around 26–29 (28); and (6) absence of dementia. The diagnosis of AD was based on the criteria of the National Institute of Neurologic and Communicative Disorders and Stroke (29). Patients with a CDR score of 1 and an MMSE score around 20–25 were regarded as early-stage AD and were recruited into the study.

The study was designed and carried out in accordance with the principles of the Declaration of Helsinki and with approval from the Ethics Review Board of Chang Gung Memorial Hospital (IRB 104-4769B and IRB 104-6261B). All subjects gave written informed consent.

### Metabolomic Analysis

Targeted metabolomic analysis of serum samples was performed using the Biocrates Absolute-IDQ P180 kit (Life Science AG, Innsbruck, Austria). The serum samples were processed as per the manufacturer instructions and analyzed on a triple-quadrupole mass spectrometer (Waters, Milford, CT, USA). As part of the quality control, three concentrations of quality controls were included in the kit. Targeted metabolic data were analyzed by TargetLynx (Waters, Milford, CT, USA), and MetIDQ software (Biocrates, Innsbruck, Austria) was applied to integrate the metabolites by automated calculation of metabolite concentrations. Metabolites with the concentration below the limit of detection were excluded.

### Statistical Analysis

Continuous variables were expressed as mean ± standard deviation (SD) if the values were normally distributed or median and interquartile range if the values were not normally distributed. Categorical variables were expressed as a number, or percentage, for each item. The MCI and early-stage AD patient groups were compared using chi-square, Mann–Whitney *U*, or Student *t*-test. To maximize identification of differences in metabolic profiles between groups, the orthogonal-projection-to-latent-structure–discriminant-analysis (OPLS-DA) model was applied using the SIMCA-P software (version 13.0, Umetrics AB, Umea, Sweden). The variable importance in the projection (VIP) value of each variable in the model was calculated to indicate its contribution to the classification. A higher VIP value represented a stronger contribution to discrimination among groups. VIP > 1.5 were considered significantly different. All statistical analyses were two-sided and performed using IBM SPSS statistics 19.0 software (Armonk, NY, USA) for Windows. A *P* < 0.05 was considered significant.

## Results

### Patient Characteristics

A total of 80 participants included 40 MCI and 40 early-stage AD patients. The characteristics and laboratory data of the included patients were shown in Table 1. The early-stage AD patients were significantly older than the MCI patients (*P* < 0.001). The durations from symptom onset to diagnosis were significantly longer in early-stage AD patients as compared with MCI patients (*P* < 0.001). Upon comorbidities, the prevalence of hyperlipidemia was significantly higher in MCI patients as compared with early-stage AD patients (*P* = 0.03). The percentage of hypnotic drug usage was significantly higher in MCI patients (*P* = 0.017). According to laboratory data, early-stage AD patients had lower levels of hemoglobin, alanine transaminase, and total cholesterol as compared with MCI patients (*P* = 0.018, < 0.001, and 0.006, respectively).

**Table 1:** Demographic characteristics of mild cognitive impairment and early-stage Alzheimer's disease patients.

	**Mild cognitive impairment (*n* = 40)**	**Early-stage Alzheimer's disease (*n* = 40)**	**Odds ratio (95% CI)**	***P*-value**
Age (years)	68 (67–72)	77 (73–81)		<0.001^*^
Female	44 (67.7%)	25 (65.8%)	0.83 (0.36–1.91)	0.415
Duration of symptoms (months)	12 (6–24)	24 (12–36)		<0.001^*^
MMSE	28 (26–29)	22 (20–24)		<0.001^*^
CDR	0.5 (0.5–0.5)	0.5 (0.5–0.5)		<0.001^*^
CASI	86 (80–91)	65 (60–72)		0.005^*^
Comorbidity				
Hypertension	33 (50.8%)	21 (55.3%)	0.84 (0.37–1.86)	0.407
Diabetes mellitus	11 (16.9%)	11 (28.9%)	0.50 (0.19–1.30)	0.118
Hyperlipidemia	36 (55.4%)	13 (34.2%)	2.39 (1.04–5.47)	0.03^*^
Coronary artery disease	9 (13.8%)	5 (13.2%)	1.06 (0.33–3.43)	0.586
Chronic kidney disease	1 (1.5%)	3 (7.9%)	0.18 (0.02–1.82)	0.141
Gout	3 (4.8)	4 (10.5)	0.41 (0.09–1.95)	0.225
Insomnia	24 (36.9%)	10 26.3%)	1.64 (0.68–3.95)	0.188
Hypnotic drug use	22 (33.8%)	5 (13.2%)	3.38 (1.16–9.86)	0.017^*^
Antidepressant use	4 (6.2%)	1 (2.6%)	2.43 (0.26–22.5)	0.388
Laboratory data				
TSH, nIU/ml	1.78 (1.07–2.34)	1.42 (1.00–2.51)		0.420
Free T4, mg/dl	1.14 (0.99–1.26)	1.13 (1.02–1.29)		0.940
Cortisol, μg/dl	9.5 (6.9–12.5)	9.1 (6.4–10.7)		0.146
Vitamin B12, pg/ml	610.3 (458.3–1,000.45)	593.95 (436.95–1,242.5)		0.954
Folate, ng/ml	9.64 (7.28–12.93)	10.97 (7.65–16.42)		0.264
WBC, 1,000/μl	5,700 (4,950–7,000)	6,150 (5,275–7,850)		0.270
Hemoglobin, g/dl	13.6 (12.6–14.5)	13.1 (12.1–13.6)		0.018^*^
Platelet, 1,000/μl	217.0 ± 54.4	220.3 ± 59.6		0.774
Sugar, mg/dl	102 (93–111)	101 (92.5–125.3)		0.523
Glycohemoglobin, g/dl	5.8 (5.6–6.2)	5.95 (5.70–6.35)		0.153
AST, U/L	23 (20–29)	23 (18–27)		0.409
ALT, U/L	23 (19–29)	16 (14–23)		<0.001^*^
Total cholesterol, mg/dl	203 (180.5–233)	182 (153.5–204)		0.006^*^
Triglyceride, mg/dl	123 (87.5–178)	99 (66.5–130.8)		0.051
LDL-C, mg/dl	119 (107–146)	111 (91–134)		0.066
HDL-C, mg/dl	47 (41–59)	48 (40–58)		0.632
Uric acid, mg/dl	6.0 (5.2–6.5)	5.9 (5.0–6.2)		0.425
hs-CRP, mg/L	0.8 (0.3–2.0)	0.8 (0.2–2.4)		0.891

*CI, confidence interval; MMSE, Mini-Mental Status Examination; CDR, Clinical Dementia Rating; CASI, Cognitive Abilities Screening Instrument; TSH, thyrotropin; T4, thyroxine; WBC, white blood cells; AST, aspartate aminotransferase; ALT, alanine transaminase; BUN, blood urea nitrogen; LDL-C, low-density lipoprotein cholesterol; HDL-C, high-density lipoprotein cholesterol; hs-CRP, high-sensitivity C-reactive protein*.

*Data are presented as mean ± standard deviation, median (interquartile range), or n (%)*.

* *P < 0.05, Student t, Mann–Whitney U, or chi-square test*.

### Serum Metabolomics Analysis

**Figure 1:**
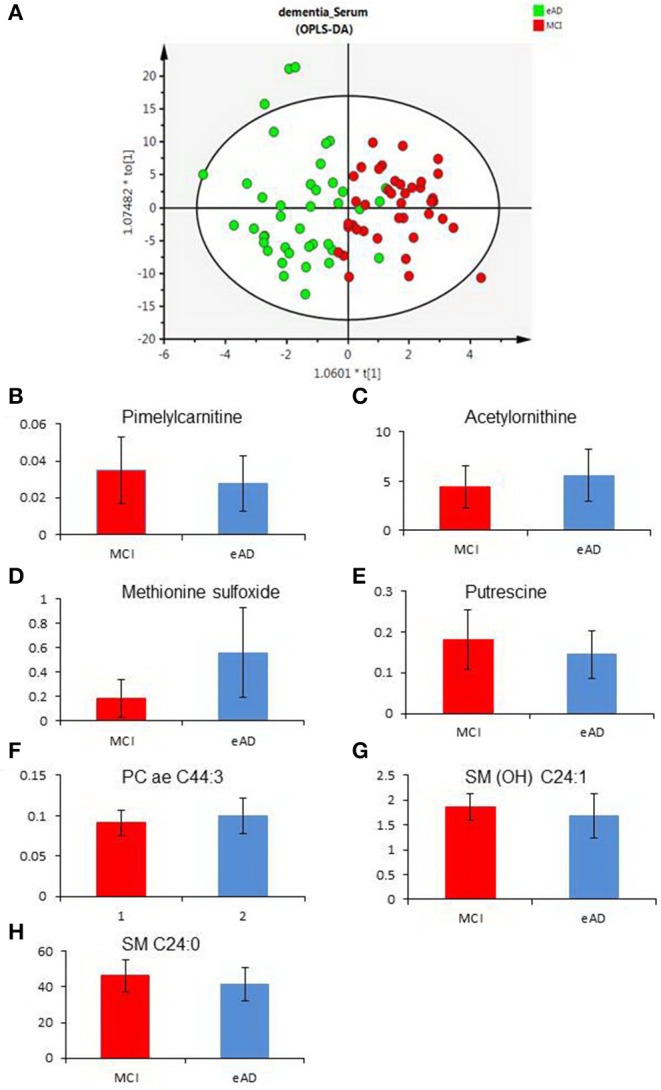
The differences of serum metabolomic profiles between mild cognitive impairment (MCI) and early-stage Alzheimer's disease (eAD) patients. **(A)** The orthogonal-projection-to-latent-structure–discriminant-analysis (OPLS-DA) score plots show the considerable separation between 40 MCI and 40 eAD patients. **(B**–**H)** Plasma levels of metabolites compared between MCI and eAD patients (*P* < 0.05). **(B–H)** Were pimelylcarnitine, acetylornithine, methionine sulfoxide, putrescine, PC ae C44:3, SM (OH) C24:1, and SM C24:0.

To evaluate the differences of serum metabolites between MCI and early-stage AD patients, serum from 40 MCI and 40 early-stage AD patients were subject to targeted metabolite analysis, and datasets were analyzed by the OPLS-DA model. The OPLS-DA score plots demonstrated considerable separation between the MCI and early-stage AD patients (Figure 1A). The metabolites responsible for the discrimination between these two groups (those with VIP > 1.5) are listed in Table 2. The levels of pimelylcarnitine, putrescine, SM (OH) C24:1, and SM C24:0 were significantly lower, whereas the levels of acetylornithine, methionine sulfoxide (Met-SO), and PC ae C44:3 were significantly higher in early-stage AD patients as compared with MCI patients (Table 2, Figures 1B–H).

**Table 2:** Statistical analysis of targeted metabolites between mild cognitive impairment and early-stage Alzheimer's disease patients.

**Metabolites, μM**	**VIP score**	**MCI**	**eAD**	***P*-value**	**AUC (95% CI)**
Pimelylcarnitine	1.98	0.035 ± 0.018	0.028 ± 0.015	0.0355	0.640 (0.516–0.764)
Acetylornithine	2.03	4.398 ± 2.147	5.584 ± 2.648	0.0309	0.654 (0.532–0.776)
Methionine sulfoxide	2.69	0.183 ± 0.262	0.561 ± 0.756	0.0044	0.630 (0.507–0.753)
Putrescine	2.30	0.182 ± 0.073	0.145 ± 0.058	0.0141	0.665 (0.545–0.784)
PC ae C44:3	1.98	0.091 ± 0.016	0.100 ± 0.022	0.0356	0.622 (0.50–0.745)
SM (OH) C24:1	1.94	1.862 ± 0.273	1.689 ± 0.443	0.0396	0.664 (0.542–0.787)
SM C24:0	2.12	46.258 ± 8.866	41.515 ± 9.489	0.0236	0.656 (0.535–0.777)

*VIP, variable importance in the projection; MCI, mild cognitive impairment; eAD, early-stage Alzheimer's disease; PC ae C44:3, phosphatidylcholine acyl-alkyl C44:3; SM (OH) C24:1, hydroxysphingomyeline; SM C24:0, sphingomyelin C24:0; receiver operating characteristics (ROC) curves with area under the curve (AUC) of single metabolites to discriminate MCI and early-stage AD patients*.

*Values are mean ± SD*.

**Figure 2:**
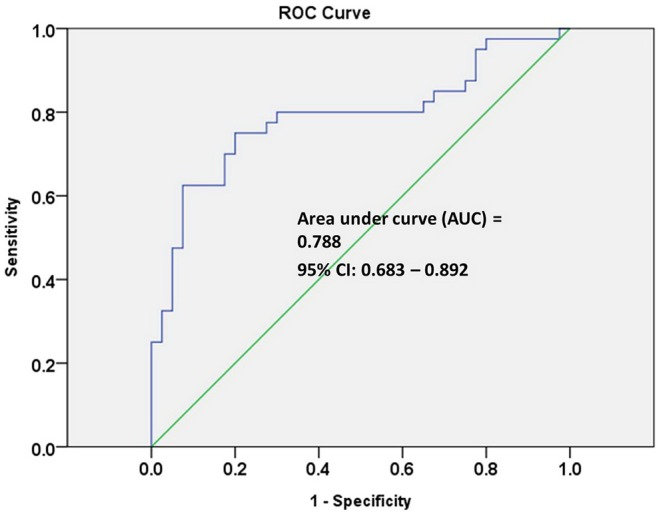
Receiver operating characteristic (ROC) curve analysis of three combined lipid metabolites of SM (OH) C24:1, SM C24:0, and PC ae C44:3 discriminate early-stage AD patients from MCI patients.

The receiver operating characteristic (ROC) curve analysis of seven individual metabolites did not show good discrimination between MCI and early-stage AD patients [area under the curve (AUC) demonstrated in Table 2]. ROC curve analysis of a combination of these seven metabolites showed good discrimination of early-stage AD and MCI patients (AUC = 0.811). However, ROC curve analysis of a combination of three lipid metabolites [SM (OH) C24:1, SM C24:0, and PC ae C44:3] showed an acceptable discrimination between MCI and early-stage AD patients (AUC = 0.788, Figure 2). The finding supported the importance of dysregulation of brain lipid metabolisms in cognitive decline.

## Discussion

In the current study, we found that the levels of several serum metabolites had significant differences between patients of MCI and early-stage AD, which might be potential biomarkers for distinguishing MCI patients who will progress to early-stage AD from stable MCI patients. The targeted metabolite analysis using the Absolute-IDQ P180 kit revealed that the levels of pimelylcarnitine, putrescine, SM (OH) C24:1, and SM C24:0 were significantly lower, whereas the levels of acetylornithine, Met-SO, and PC ae C44:3 were significantly higher in serums of early-stage AD patients as compared with MCI patients.

Previous studies have demonstrated that the metabolites of cerebrospinal fluid could predict dementia development. However, the procedure of collecting cerebrospinal fluid is relatively invasive (30, 31). The diagnostic value of less invasive biomarkers, such as metabolites of blood or saliva samples, remains controversial. Previous blood metabolite studies of AD suggested that some lipids (32, 33) or metabolites (10, 34) could predict the progress of AD. In addition, Zheng et al. found that cognitively healthy adults and MCI patients could be differentiated with each other by the analyses of metabolites identified from saliva samples (14). However, other studies did not reveal a clear group separation for saliva samples among dementia and normal patients but found significant differences for serum samples (16). This finding suggests that blood samples are more suitable for non-invasive biomarker discovery of AD as compared with saliva.

Our results showed that the serum level of pimelylcarnitine, one kind of acylcarnitine, was significantly lower in early-stage AD patients as compared with MCI patients. Pimelylcarnitine had been reported to be associated with sleep deprivation (35), migraine (36), and type II diabetes mellitus (37). Acylcarnitines are formed from the conversion of acyl-CoA species by carnitine palmitoyltransferase (CPT) 1 (38). Then acylcarnitines are transported into the mitochondrial matrix by the carnitine acylcarnitine translocase, a mitochondrial inner membrane transporter. Finally, the enzyme CPT2 reconverts acylcarnitines back into free carnitine and long-chain acyl-CoAs (39). There is growing evidence that a net efflux of acylcarnitine species from the mitochondria into the cytosol and finally into plasma is crucial in the conditions of impaired fatty acid oxidation, because it could prevent the potentially toxic acyl-CoA intermediates from accumulating in the mitochondrion (40). Therefore, the changes of plasma or urinary acylcarnitine profiles have been used to detect fatty acid and amino acid oxidation disorders (41–43).

Our results found that the serum level of Met-SO was significantly higher in early-stage AD patients as compared with MCI patients. Met-SO, an oxidized form of methionine, is known to be an indicator of systemic oxidative stress since methionine is particularly susceptible to oxidization by reactive oxygen species (44, 45). To protect the cells from these toxic radicals, the Met-SO reductase system could reduce Met-SO to methionine (46). In a previous AD study, oxidative stress is one of the earliest consequences of toxic insults mediated by soluble amyloid β-protein (Aβ) oligomers (47). The Met-SO has been found to comprise 10–50% of Aβ in amyloid plaques of AD brain (48). As the oxidative stress is increased with advanced age and dementia stage while the activity of the methionine sulfoxide reductase system and other antioxidants are decreased, the level of Met-SO should be higher in more severe AD stages. This might be able to explain why the level of Met-SO is higher in early-stage AD patients when compared to MCI patients in this study.

Our results revealed that the serum level of putrescine, the precursor of spermidine, was significantly lower in early-stage AD patients as compared with MCI patients. Hydrolysis of l-arginine by arginase generates l-ornithine, which is decarboxylated by ornithine decarboxylase to form putrescine. Putrescine can also be produced by hydrolysis of agmatine, which is formed from l-arginine by arginine decarboxylase. Putrescine is alkylated by the decarboxylated *S*-adenosyl-methionine, which regularly serves as the major methyl group donor for methyl transferases (49). There is growing evidence that arginine metabolism may be associated with the development of AD and other dementia (50–52). Furthermore, in a recent study by Zhang et al., reduced urinary arginine levels were noted in patients with amnestic MCI (22).

In our results, the serum levels of SM (OH) C24:1 and SM C24:0 were significantly lower in early-stage AD patients as compared with MCI patients. In the brain, the proper balance of sphingolipids is essential for normal neuronal function, and subtle changes in sphingolipid balance may be intimately involved in neurodegenerative diseases, including AD. SMs are major components of cell membranes and are especially enriched in the central nervous system. Clinical, laboratory, and animal studies thus far suggest that perturbations in SM may contribute to the pathophysiology of AD, particularly the formation of amyloid-beta, associated amyloid plaques, and neurodegeneration (53). Previous study revealed that higher levels of SM (OH) C22:1 were significantly associated with lower risk of dementia (7). Besides, higher concentrations of three other SMs [SM C26:0, SM (OH) C22:2, and SM (OH) C24:1] might have a trend of lower prevalence of dementia, though these associations did not reach statistical significance (7). Our results are in agreement with previous findings on the link between AD and SMs (7, 53, 54). In the brain, the proper balance of sphingolipids is important for maintaining normal neuronal function. Therefore, the process of sphingolipid metabolism is altered in the early stage of AD and contributes to the neuropathological disturbances of AD, which include Aβ production, tau protein formation, and neurodegeneration (55).

We found that *N*-acetylornithine was significantly higher in early-stage AD as compared with MCI patients. *N*-Acetylornithine, an intermediate in arginine metabolism, is a minor component of deproteinized human blood (56). It has been linked to chronic kidney disease and been regarded as a potential biomarker for kidney function in humans since a previous study found that higher levels of *N*-acetylornithine were associated with lower estimated glomerular filtration rate (57). However, no previous study has linked *N*-acetylornithine to neurodegenerative diseases except one recent study revealing that there is a relationship between decreased *N*-acetylornithine level and haloperidol exposure (58). Therefore, a causal relationship between increased *N*-acetylornithine levels and early-stage AD, as observed in our study, is difficult to speculate upon.

In addition, the level of PC ae C44:3, a metabolite of glycerophospholipids, was significantly higher in early-stage AD patients as compared with MCI patients. Glycerophospholipids, one kind of brain lipids, constitute a varied group of molecules with important brain functions (59). Several studies suggest that the abnormal metabolism of glycerophospholipids is associated with some important features of AD, such as neuroinflammation, neuronal injury, and neurodegeneration (60–62). Previous studies also found that several glycerophospholipids were significantly increased in the cerebrospinal fluid (CSF) samples with AD-like pathology (63, 64). Therefore, due to the importance of these brain lipids in the cognitive decline, the three combined lipid metabolites of SM (OH) C24:1, SM C24:0, and PC ae C44:3 will result in an acceptable discrimination between early-stage AD patients and MCI patients in our study.

This study has several limitations. First, the sample size was relatively small. Second, this was a cross-sectional study to compare the metabolomics between MCI and early-stage AD patients. Long-term follow-up was indicated to realize which metabolites could predict MCI or AD before the onsets, but it took many years. Our findings may serve as a pilot study. Third, there was no age-matched control group in our study. The lack of a control group was mainly due to the strict exclusion criteria of our study. Fourth, the interference of hypnotic drugs on serum metabolomics was unknown, since no published studies had discussed about the effects of different hypnotics drugs on metabolic profiles in patients with cognitive impairment. Even with these limitations, we believed the presented results might be the base of future multicentric studies, with larger samples and longer follow-up.

## Conclusions

In this study, we identified the differences of serum metabolites between patients of MCI and early-stage AD. The changes of these metabolites reflect increased severity of neuronal injury and neurodegeneration in early-stage AD than MCI. The positive findings from the present serum metabolomics analyses, together with other recent findings, indicate that the serum metabolomics analysis may help to distinguish MCI patients from early-stage AD patients clinically. Long-term follow-up studies with larger samples will be performed in the future to see if these metabolites can predict the MCI progression to AD.

## Data Availability Statement

The datasets used and/or analyzed during the current study are available from the corresponding author on reasonable request.

## Ethics Statement

The studies involving human participants were reviewed and approved by Ethics Review Board of Chang Gung Memorial Hospital. The patients/participants provided their written informed consent to participate in this study.

## Author Contributions

W-CW, W-YH, and K-HC: conceptualization. K-HC, H-YT, and M-LC: methodology. H-YT: software. K-HC, H-YT, and M-LC: validation. W-YH and H-YT: formal analysis. W-YH: data curation. W-CW and W-YH: writing—original draft preparation. K-HC and W-YH: writing—review and editing. K-HC: supervision. W-CW and W-YH: project administration. W-CW: funding acquisition. All authors read and approved the final version of the manuscript and agreed to be accountable for all aspects of the work.

### Conflict of Interest

The authors declare that the research was conducted in the absence of any commercial or financial relationships that could be construed as a potential conflict of interest.

## References

[1] Hardy J Selkoe DJ. The amyloid hypothesis of Alzheimer's disease: progress and problems on the road to therapeutics. Science. (2002) 297:353–6. 10.1126/science.1072994 12130773

[2] Qiu C De Ronchi D Fratiglioni L. The epidemiology of the dementias: an update. Curr Opin Psychiatry. (2007) 20:380–5. 10.1097/YCO.0b013e32816ebc7b 17551353

[3] Petersen RC. Mild cognitive impairment as a diagnostic entity. J Intern Med. (2004) 256:183–94. 10.1111/j.1365-2796.2004.01388.x 15324362

[4] Petersen RC Stevens JC Ganguli M Tangalos EG Cummings JL DeKosky ST. Practice parameter: early detection of dementia: mild cognitive impairment (an evidence-based review). Report of the quality standards subcommittee of the American Academy of Neurology. Neurology. (2001) 56:1133–42. 10.1212/WNL.56.9.1133 11342677

[5] Gauthier S Reisberg B Zaudig M Petersen RC Ritchie K Broich K. Mild cognitive impairment. Lancet. (2006) 367:1262–70. 10.1016/S0140-6736(06)68542-5 16631882

[6] Klavins K Koal T Dallmann G Marksteiner J Kemmler G Humpel C. The ratio of phosphatidylcholines to lysophosphatidylcholines in plasma differentiates healthy controls from patients with Alzheimer's disease and mild cognitive impairment. Alzheimers Dement. (2015) 1:295–302. 10.1016/j.dadm.2015.05.003 26744734 PMC4700585

[7] Li D Misialek JR Boerwinkle E Gottesman RF Sharrett AR Mosley TH Plasma phospholipids and prevalence of mild cognitive impairment and/or dementia in the ARIC Neurocognitive Study (ARIC-NCS). Alzheimers Dement. (2016) 3:73–82. 10.1016/j.dadm.2016.02.008 PMC4925799 27408938

[8] Marksteiner J Blasko I Kemmler G Koal T Humpel C. Bile acid quantification of 20 plasma metabolites identifies lithocholic acid as a putative biomarker in Alzheimer's disease. Metabolomics. (2018) 14:1. 10.1007/s11306-017-1297-5 29249916 PMC5725507

[9] Oberacher H Arnhard K Linhart C Diwo A Marksteiner J Humpel C. Targeted metabolomic analysis of soluble lysates from platelets of patients with mild cognitive impairment and alzheimer's disease compared to healthy controls: is PC aeC40:4 a promising diagnostic tool? J Alzheimers Dis. (2017) 57:493–504. 10.3233/JAD-160172 28269764

[10] Oresic M Hyotylainen T Herukka SK Sysi-Aho M Mattila I Seppanan-Laakso T. Metabolome in progression to Alzheimer's disease. Transl Psychiatry. (2011) 1:e57. 10.1038/tp.2011.55 22832349 PMC3309497

[11] St John-Williams L Blach C Toledo JB Rotroff DM Kim S Klavins K Targeted metabolomics and medication classification data from participants in the ADNI1 cohort. Sci Data. (2017) 4:170140 10.1038/sdata.2017.140 29039849 PMC5644370

[12] Wood PL Locke VA Herling P Passaro A Vigna GB Volpato S. Targeted lipidomics distinguishes patient subgroups in mild cognitive impairment (MCI) and late onset Alzheimer's disease (LOAD). BBA Clin. (2016) 5:25–8. 10.1016/j.bbacli.2015.11.004 27051586 PMC4802395

[13] Yilmaz A Geddes T Han B Bahado-Singh RO Wilson GD Imam K. Diagnostic biomarkers of Alzheimer's disease as identified in saliva using 1H NMR-based metabolomics. J Alzheimers Dis. (2017) 58:355–9. 10.3233/JAD-161226 28453477

[14] Zheng J Dixon RA Li L. Development of isotope labeling LC-MS for human salivary metabolomics and application to profiling metabolome changes associated with mild cognitive impairment. Anal Chem. (2012) 84:10802–11. 10.1021/ac3028307 23150892 PMC3526113

[15] Kallo G Emri M Varga Z Ujhelyi B Tozser J Csutak A. Changes in the chemical barrier composition of tears in Alzheimer's disease reveal potential tear diagnostic biomarkers. PLoS ONE. (2016) 11:e0158000. 10.1371/journal.pone.0158000 27327445 PMC4915678

[16] Tsuruoka M Hara J Hirayama A Sugimoto M Soga T Shankle WR. Capillary electrophoresis-mass spectrometry-based metabolome analysis of serum and saliva from neurodegenerative dementia patients. Electrophoresis. (2013) 34:2865–72. 10.1002/elps.201300019 23857558

[17] Blennow K. CSF biomarkers for Alzheimer's disease: use in early diagnosis and evaluation of drug treatment. Expert Rev Mol Diagn. (2005) 5:661–72. 10.1586/14737159.5.5.661 16149870

[18] Humpel C. Identifying and validating biomarkers for Alzheimer's disease. Trends Biotechnol. (2011) 29:26–32. 10.1016/j.tibtech.2010.09.007 20971518 PMC3016495

[19] van der Greef J Stroobant P van der Heijden R. The role of analytical sciences in medical systems biology. Curr Opin Chem Biol. (2004) 8:559–65. 10.1016/j.cbpa.2004.08.013 15450501

[20] Jiang Y Zhu Z Shi J An Y Zhang K Wang Y. Metabolomics in the development and progression of dementia: a systematic review. Front Neurosci. (2019) 13:343. 10.3389/fnins.2019.00343 31031585 PMC6474157

[21] Fleszar MG Wisniewski J Zboch M Diakowska D Gamian A Krzystek-Korpacka M. Targeted metabolomic analysis of nitric oxide/L-arginine pathway metabolites in dementia: association with pathology, severity, and structural brain changes. Sci Rep. (2019) 9:13764. 10.1038/s41598-019-50205-0 31551443 PMC6760237

[22] Zhang YQ Tang YB Dammer E Liu JR Zhao YW Zhu L. Dysregulated urinary arginine metabolism in older adults with amnestic mild cognitive impairment. Front Aging Neurosci. (2019) 11:90. 10.3389/fnagi.2019.00090 31105552 PMC6492563

[23] Kim M Snowden S Suvitaival T Ali A Merkler DJ Ahmad T. Primary fatty amides in plasma associated with brain amyloid burden, hippocampal volume, and memory in the European Medical Information Framework for Alzheimer's Disease biomarker discovery cohort. Alzheimers Dement. (2019) 15:817–27. 10.1016/j.jalz.2019.03.004 31078433 PMC6849698

[24] Tombaugh TN McIntyre NJ. The mini-mental state examination: a comprehensive review. J Am Geriatr Soc. (1992) 40:922–35. 10.1111/j.1532-5415.1992.tb01992.x 1512391

[25] Berg L. Clinical dementia rating (CDR). Psychopharmacol Bull. (1988) 24:637–9. 3249765

[26] Teng EL Hasegawa K Homma A Imai Y Larson E Graves A. The Cognitive Abilities Screening Instrument (CASI): a practical test for cross-cultural epidemiological studies of dementia. Int Psychogeriatr. (1994) 6:45–58. 10.1017/S1041610294001602 8054493

[27] Petersen RC Doody R Kurz A Mohs RC Morris JC Rabins PV. Current concepts in mild cognitive impairment. Arch Neurol. (2001) 58:1985–92. 10.1001/archneur.58.12.1985 11735772

[28] Koontz J Baskys A. Effects of galantamine on working memory and global functioning in patients with mild cognitive impairment: a double-blind placebo-controlled study. Am J Alzheimers Dis Other Demen. (2005) 20:295–302. 10.1177/153331750502000502 16273995 PMC10833248

[29] McKhann G Drachman D Folstein M Katzman R Price D Stadlan EM. Clinical diagnosis of Alzheimer's disease: report of the NINCDS-ADRDA Work Group under the auspices of department of health and human services task force on Alzheimer's disease. Neurology. (1984) 34:939–44. 10.1212/WNL.34.7.939 6610841

[30] Gustafson DR Skoog I Rosengren L Zetterberg H Blennow K. Cerebrospinal fluid beta-amyloid 1-42 concentration may predict cognitive decline in older women. J Neurol Neurosurg Psychiatry. (2007) 78:461–4. 10.1136/jnnp.2006.100529 17098843 PMC2117838

[31] Mattsson N Rosen E Hansson O Andreasen N Parnetti L Jonsson M. Age and diagnostic performance of Alzheimer disease CSF biomarkers. Neurology. (2012) 78:468–76. 10.1212/WNL.0b013e3182477eed 22302554 PMC3280049

[32] Han G Wang J Zeng F Feng X Yu J Cao HY. Characteristic transformation of blood transcriptome in Alzheimer's disease. J Alzheimers Dis. (2013) 35:373–86. 10.3233/JAD-121963 23411692

[33] Proitsi P Kim M Whiley L Simmons A Sattlecker M Velayudhan L. Association of blood lipids with Alzheimer's disease: a comprehensive lipidomics analysis. Alzheimers Dement. (2017) 13:140–51. 10.1016/j.jalz.2016.08.003 27693183

[34] Mapstone M Cheema AK Fiandaca MS Zhong X Mhyre TR MacArthur LH. Plasma phospholipids identify antecedent memory impairment in older adults. Nat Med. (2014) 20:415–8. 10.1038/nm.3466 24608097 PMC5360460

[35] Davies SK Ang JE Revell VL Holmes B Mann A Robertson FP. Effect of sleep deprivation on the human metabolome. Proc Natl Acad Sci USA. (2014) 111:10761–6. 10.1073/pnas.1402663111 25002497 PMC4115565

[36] Ren C Liu J Zhou J Liang H Wang Y Sun Y. Lipidomic analysis of serum samples from migraine patients. Lipids Health Dis. (2018) 17:22. 10.1186/s12944-018-0665-0 29394939 PMC5797421

[37] van Valkengoed IGM Argmann C Ghauharali-van der Vlugt K Aerts J Brewster LM Peters RJG. Ethnic differences in metabolite signatures and type 2 diabetes: a nested case-control analysis among people of South Asian, African and European origin. Nutr Diabetes. (2017) 7:300. 10.1038/s41387-017-0003-z 29259157 PMC5865542

[38] Ramsay RR Gandour RD van der Leij FR. Molecular enzymology of carnitine transfer and transport. Biochim Biophys Acta. (2001) 1546:21–43. 10.1016/S0167-4838(01)00147-9 11257506

[39] Schooneman MG Vaz FM Houten SM Soeters MR. Acylcarnitines: reflecting or inflicting insulin resistance? Diabetes. (2013) 62:1–8. 10.2337/db12-0466 23258903 PMC3526046

[40] Violante S Ijlst L Ruiter J Koster J van Lenthe H Duran M. Substrate specificity of human carnitine acetyltransferase: Implications for fatty acid and branched-chain amino acid metabolism. Biochim Biophys Acta. (2013) 1832:773–9. 10.1016/j.bbadis.2013.02.012 23485643

[41] Adams SH Hoppel CL Lok KH Zhao L Wong SW Minkler PE. Plasma acylcarnitine profiles suggest incomplete long-chain fatty acid beta-oxidation and altered tricarboxylic acid cycle activity in type 2 diabetic African-American women. J Nutr. (2009) 139:1073–81. 10.3945/jn.108.103754 19369366 PMC2714383

[42] Giesbertz P Ecker J Haag A Spanier B Daniel H. An LC-MS/MS method to quantify acylcarnitine species including isomeric and odd-numbered forms in plasma and tissues. J Lipid Res. (2015) 56:2029–39. 10.1194/jlr.D061721 26239049 PMC4583086

[43] Wang-Sattler R Yu Z Herder C Messias AC Floegel A He Y. Novel biomarkers for pre-diabetes identified by metabolomics. Mol Syst Biol. (2012) 8:615. 10.1038/msb.2012.43 23010998 PMC3472689

[44] Shringarpure R Davies KJ. Protein turnover by the proteasome in aging and disease. Free Radic Biol Med. (2002) 32:1084–9. 10.1016/S0891-5849(02)00824-9 12031893

[45] Stadtman ER Van Remmen H Richardson A Wehr NB Levine RL. Methionine oxidation and aging. Biochim Biophys Acta. (2005) 1703:135–40. 10.1016/j.bbapap.2004.08.010 15680221

[46] Oien DB Moskovitz J. Substrates of the methionine sulfoxide reductase system and their physiological relevance. Curr Top Dev Biol. (2008) 80:93–133. 10.1016/S0070-2153(07)80003-2 17950373

[47] Hirai K Aliev G Nunomura A Fujioka H Russell RL Atwood CS. Mitochondrial abnormalities in Alzheimer's disease. J Neurosci. (2001) 21:3017–23. 10.1523/JNEUROSCI.21-09-03017.2001 11312286 PMC6762571

[48] Jiang B Moskovitz J. The functions of the mammalian methionine sulfoxide reductase system and related diseases. Antioxidants. (2018) 7:122. 10.3390/antiox7090122 30231496 PMC6162418

[49] Tsikas D Erik H Gorig B Spermidine for a long, dementia-free life? Glob J Pharmaceu Sci. (2017) 2:001–8. 10.19080/GJPPS.2017.02.555576

[50] Mousavi M Jonsson P Antti H Adolfsson R Nordin A Bergdahl J. Serum metabolomic biomarkers of dementia. Dement Geriatr Cogn Dis Extra. (2014) 4:252–62. 10.1159/000364816 25177334 PMC4132238

[51] Pan X Cunningham EL Passmore AP McGuinness B McAuley DF Beverland D. Cerebrospinal fluid spermidine, glutamine and putrescine predict postoperative delirium following elective orthopaedic surgery. Sci Rep. (2019) 9:4191. 10.1038/s41598-019-40544-3 30862889 PMC6414730

[52] Yi J Horky LL Friedlich AL Shi Y Rogers JT Huang X. L-arginine and Alzheimer's disease. Int J Clin Exp Pathol. (2009) 2:211–38. 19079617 PMC2600464

[53] Mielke MM Haughey NJ Bandaru VV Weinberg DD Darby E Zaidi N. Plasma sphingomyelins are associated with cognitive progression in Alzheimer's disease. J Alzheimers Dis. (2011) 27:259–69. 10.3233/JAD-2011-110405 21841258 PMC3218198

[54] Mielke MM Bandaru VV Haughey NJ Rabins PV Lyketsos CG Carlson MC. Serum sphingomyelins and ceramides are early predictors of memory impairment. Neurobiol Aging. (2010) 31:17–24. 10.1016/j.neurobiolaging.2008.03.011 18455839 PMC2783210

[55] Mielke MM Haughey NJ Could plasma sphingolipids be diagnostic or prognostic biomarkers for Alzheimer's disease? Clin Lipidol. (2012) 7:525–36. 10.2217/clp.12.59 23606909 PMC3627378

[56] Armstrong MD. N-delta-acetylornithine and S-methylcysteine in blood plasma. Biochim Biophys Acta. (1979) 587:638–42. 10.1016/0304-4165(79)90015-1 508804

[57] Suhre K Shin SY Petersen AK Mohney RP Meredith D Wagele B. Human metabolic individuality in biomedical and pharmaceutical research. Nature. (2011) 477:54–60. 10.1038/nature10354 21886157 PMC3832838

[58] McClay JL Vunck SA Batman AM Crowley JJ Vann RE Beardsley PM. Neurochemical metabolomics reveals disruption to sphingolipid metabolism following chronic haloperidol administration. J Neuroimmune Pharmacol. (2015) 10:425–34. 10.1007/s11481-015-9605-1 25850894 PMC4546545

[59] Fonteh AN Chiang J Cipolla M Hale J Diallo F Chirino A. Alterations in cerebrospinal fluid glycerophospholipids and phospholipase A2 activity in Alzheimer's disease. J Lipid Res. (2013) 54:2884–97. 10.1194/jlr.M037622 23868911 PMC3770101

[60] Farooqui AA Liss L Horrocks LA. Neurochemical aspects of Alzheimer's disease: involvement of membrane phospholipids. Metab Brain Dis. (1988) 3:19–35. 10.1007/BF01001351 3062351

[61] Kosicek M Hecimovic S. Phospholipids and Alzheimer's disease: alterations, mechanisms and potential biomarkers. Int J Mol Sci. (2013) 14:1310–22. 10.3390/ijms14011310 23306153 PMC3565322

[62] Soderberg M Edlund C Kristensson K Dallner G. Fatty acid composition of brain phospholipids in aging and in Alzheimer's disease. Lipids. (1991) 26:421–5. 10.1007/BF02536067 1881238

[63] Koal T Klavins K Seppi D Kemmler G Humpel C. Sphingomyelin SM(d18:1/18:0) is significantly enhanced in cerebrospinal fluid samples dichotomized by pathological amyloid-beta42, tau, and phospho-tau-181 levels. J Alzheimers Dis. (2015) 44:1193–201. 10.3233/JAD-142319 25408209 PMC4699259

[64] Wilkins JM Trushina E. Application of metabolomics in Alzheimer's disease. Front Neurol. (2017) 8:719. 10.3389/fneur.2017.00719 29375465 PMC5770363

